# Effects of acute psychosocial stress on cue‐reactivity, attentional bias and implicit associations in women with problematic social network use: An experimental study

**DOI:** 10.1111/add.70099

**Published:** 2025-06-13

**Authors:** Annica Kessling, Astrid Müller, Oliver T. Wolf, Christian J. Merz, Matthias Brand, Elisa Wegmann

**Affiliations:** ^1^ General Psychology: Cognition Faculty of Computer Science, University of Duisburg‐Essen Duisburg Germany; ^2^ Department of Psychosomatic Medicine and Psychotherapy Hannover Medical School Hannover Germany; ^3^ Department of Cognitive Psychology Institute of Cognitive Neuroscience, Faculty of Psychology, Ruhr University Bochum Bochum Germany; ^4^ Erwin L. Hahn Institute for Magnetic Resonance Imaging Essen Germany; ^5^ Center for Behavioral Addiction Research (CeBAR), Center for Translational Neuro‐ and Behavioral Sciences (C‐TNBS), University Hospital Essen, University of Duisburg‐Essen Essen Germany

**Keywords:** cognitive impairment, cognitive sets, internet‐use disorder, saliva stress measures, stress induction, structured interview

## Abstract

**Background and aims:**

Situational triggers such as acute stress may exert significant effects on behavioral execution in addictive behaviors potentially leading to increased cue‐reactivity and the expression of implicit cognitions. We measured the effects of acute stress on cue‐reactivity, attentional bias and implicit associations to stimuli related to online social networks (SN) in problematic social network use (p‐SNU) among women.

**Design, setting and participants:**

This was a mixed‐method, cross‐sectional, between‐subjects design with 135 female participants recruited in Germany who were assigned to the group with p‐SNU (*n* = 71) or the control group (*n* = 64) based on a diagnostic interview using DSM‐5 criteria for gaming disorder (applied to p‐SNU). Participants were randomly exposed to acute stress using the Trier Social Stress Test (TSST) or a placebo‐TSST.

**Measurements:**

Participants performed a Cue‐Reactivity Paradigm, Implicit Association Test and the Dot Probe Paradigm with SN‐related stimuli.

**Findings:**

Acute stress led to increased subjective urge to use social networks in both groups [TSST: mean (M) = 2.26, standard deviation (SD) = 0.92, placebo‐TSST: M = 2.08, SD = 0.96, *F*(1,131) = 6.820, *P* = 0.01, ηp^2^ = 0.029]. In the placebo‐TSST condition, the p‐SNU group showed increased subjective arousal (p‐SNU: M = 2.39, SD = 0.74; control group: M = 1.79, SD = 0.90, t_70_ = 2.55, *P* = 0.013, │*d*│ = 0.30) and urge (p‐SNU: M = 2.49, SD = 0.84; control group: M = 1.60, SD = 0.88, t_70_ = 5.40,  *P* < 0.001, │*d*│ = 0.58) and the control group showed increased attentional bias (p‐SNU: M = ‐1.75, SD = 16.11; control group: M = 6.43, SD = 15.3, t_67_ = 2.136, *P* = 0.036, │*d*│ = 0.52). No group difference was found regarding the effects of acute stress on implicit associations to SN‐related stimuli or an interaction effect of subjective urge and stress on implicit cognitions.

**Conclusions:**

Among women in Germany, acute stress appears to lead to an increased subjective urge for the use of social networks. Women with problematic social network use report higher subjective urge independent of stress, whereas women with non‐problematic use report an attentional bias.

## INTRODUCTION

The ubiquitous presence and easy accessibility of (on‐line) social networks (SN) make it increasingly difficult for people to control their use and, especially in exceptional situations (e.g. under stress), to not resort to them as a quick distraction to avoid negative emotional states. The seeming habituation to these usage patterns and a predominantly negatively connoted, compensation‐driven motivation to use increases the likelihood of developing problematic social network use (p‐SNU) over time, which can have negative impact on mental health [1] and functional impairments in everyday life [2]. To date, p‐SNU has not been included as an officially recognized mental disorder in the International Classification of Diseases (ICD)‐11 (World Health Organization, 2019). The debate about recognition is very topical since many researchers advocate for its inclusion in the ICD‐11 category ‘other specified disorders due to addictive behaviors’ (6C5Y), as parallels to officially recognized addictive behaviors are being identified [3, 4]. However, p‐SNU is one of the most controversial behaviors in the category of other addictive disorders because of a lack of clear definitions, valid measurement tools and diagnostic standards, as well as specific thresholds for p‐SNU, which is also illustrated in the limited evidence for effective treatments [5].

Theoretical models such as the Interaction of Person‐Affect‐Cognition‐Execution (I‐PACE) model [6] describe the interplay of predisposing variables, external and internal triggers as well as affective and cognitive processes such as cue‐reactivity, craving and implicit cognitions resulting in the development and maintenance of behavioral addictions. The I‐PACE model also emphasizes how situational triggers such as acute stress can contribute to the subjective urge and cue‐induced craving for use [6]. Acute stress can lead to the activation of the sympathetic nervous system and the hypothalamic–pituitary–adrenal (HPA) axis, which can cause the experience of both physical reactions such as an increased heart rate, blood flow or dilated pupils [7] and a focus on potential rewards leading to riskier decision‐making [8] or impaired self‐control [9]. The activation of autonomic and endocrine responses is often a physiological concomitant of negative feelings and can increase sensitization of the motivational systems by shifting from positive to negative reinforcement through the sensitization of the amygdala by corticotropin‐releasing factors [10, 11]. In this way, these responses help the individual cope with the physiological demands of immediate negative feelings, but do not directly alleviate the emotional distress associated with them. It is, therefore, assumed that stress‐related substance use results from the interaction between stress and the reward systems and appears to promote stress‐related relapses [12]. Consequently, acute stress constitutes a decisive factor in increasing vulnerability to addiction‐related stimuli and intensifying cravings for harmful behavior, which increases the risk of relapse [13, 14].

Higher susceptibilities to acute stress are found in studies on substance use disorders [10] and various problematic on‐line behaviors, which are reflected in higher cortisol levels and urge ratings for gaming [15] and gambling [16]. Additionally, higher subjectively rated stress was associated with higher symptom severity of compulsive buying shopping [17] or p‐SNU [18]. To cope with stress, individuals may learn to perform their behavior in a seemingly automatic and stimulus‐driven manner. This could favor a higher sensitivity to addiction‐related stimuli through the activation of reward‐related brain regions and can trigger cue‐reactivity and craving [16, 19]. Increased cue‐reactivity and craving to addiction‐related cues have been found in studies on substance use disorders [20], gaming [21, 22], buying shopping disorder [23] and p‐SNU [24, 25, 26].

The physiological effects of stress, in turn, are reflected in the positive association between cortisol levels in problematic on‐line pornography use and the activation of the reward system in response to sexual stimuli [27]. However, there are also studies illustrating no significant changes in experienced craving following stress induction in individuals with gambling disorder [16, 28]. A recent review on stress in compulsive buying‐shopping disorder highlights the need to investigate the effects of experimentally induced stress further. It is the result of limited and heterogeneous empirical evidence. Moreover, studies with clinical samples and systematic group comparisons between non‐problematic users and individuals with problematic use are highly needed [29].

Going a step further within the I‐PACE model, it is assumed that cue‐induced craving promotes the development of specific implicit cognitive processes that can manifest themselves in a higher attentional bias [30, 31] and positive implicit associations [32, 33] toward addiction‐related stimuli. Increased attentional bias or positive implicit associations with addiction‐related stimuli were shown in substance use disorders [34] and various problematic on‐line behaviors [35, 36, 37]. In contrast, the number of studies investigating the expression of attentional biases or implicit associations in p‐SNU is limited, the results are heterogeneous and only a few clinical samples have been investigated to date [38]. However, the relationship between craving and implicit cognitions is supported by theoretical assumptions, according to which conditioned stimuli lead to an automatic increase in attention, which intensifies the craving and ultimately contributes to relapse [39]. In addition, a meta‐analysis reported a link between an increased attentional bias to substance‐related stimuli and immediate craving for substance use such as alcohol [40]. Implicit cognitions such as attentional bias are, therefore, a mechanism that can be reinforced by stimulus‐induced craving. Addressing the interplay of stress, craving and affective and cognitive mechanisms, Schröder and Mühlberger [41] demonstrated that acute psychosocial stress in smokers leads to increased craving and a change in implicit cognitions. In the other sub‐study of the present project, no significant influence of craving on the relationship between stress response and implicit cognitions in the context of problematic on‐line buying‐shopping could be identified [42]; how these results can be transferred to p‐SNU is not yet clear.

Considering theoretical assumptions and current empirical findings, the extent to which acute stress in p‐SNU triggers the immediate urge to use SN and increases the implicit awareness of SN‐related cues has not yet been sufficiently investigated. There is also a lack of research examining the extent to which the urge to consume under stress leads to changes in the implicit cognitions of p‐SNU and how these differ from users without p‐SNU.

Therefore, the aim of this study is to examine if individuals in an acute stress condition experience higher subjective urge on confrontation with the SN‐related cues compared to individuals in a non‐stressed condition. We hypothesize that the effects of acute stress on cue‐reactivity and craving (measured by subjective arousal, urge and valence) are stronger in individuals with p‐SNU compared to individuals without p‐SNU (hypothesis 1). We also hypothesize that individuals in an acute stress condition experience higher attentional bias and positive implicit associations on confrontation with the SN‐related cues compared to non‐stressed individuals and that the effects of acute stress on attentional bias and implicit associations to SN‐related visual cues are stronger in individuals with p‐SNU than in the individuals without p‐SNU (hypothesis 2). Additionally, we aim to investigate if the effects of acute stress versus non‐stress on implicit cognitions are moderated by craving responses within the p‐SNU group. This interaction is expected to be stronger for SN‐related stimuli versus control stimuli (hypothesis 3).

## MATERIALS AND METHODS

### Procedure

**FIGURE 1:**
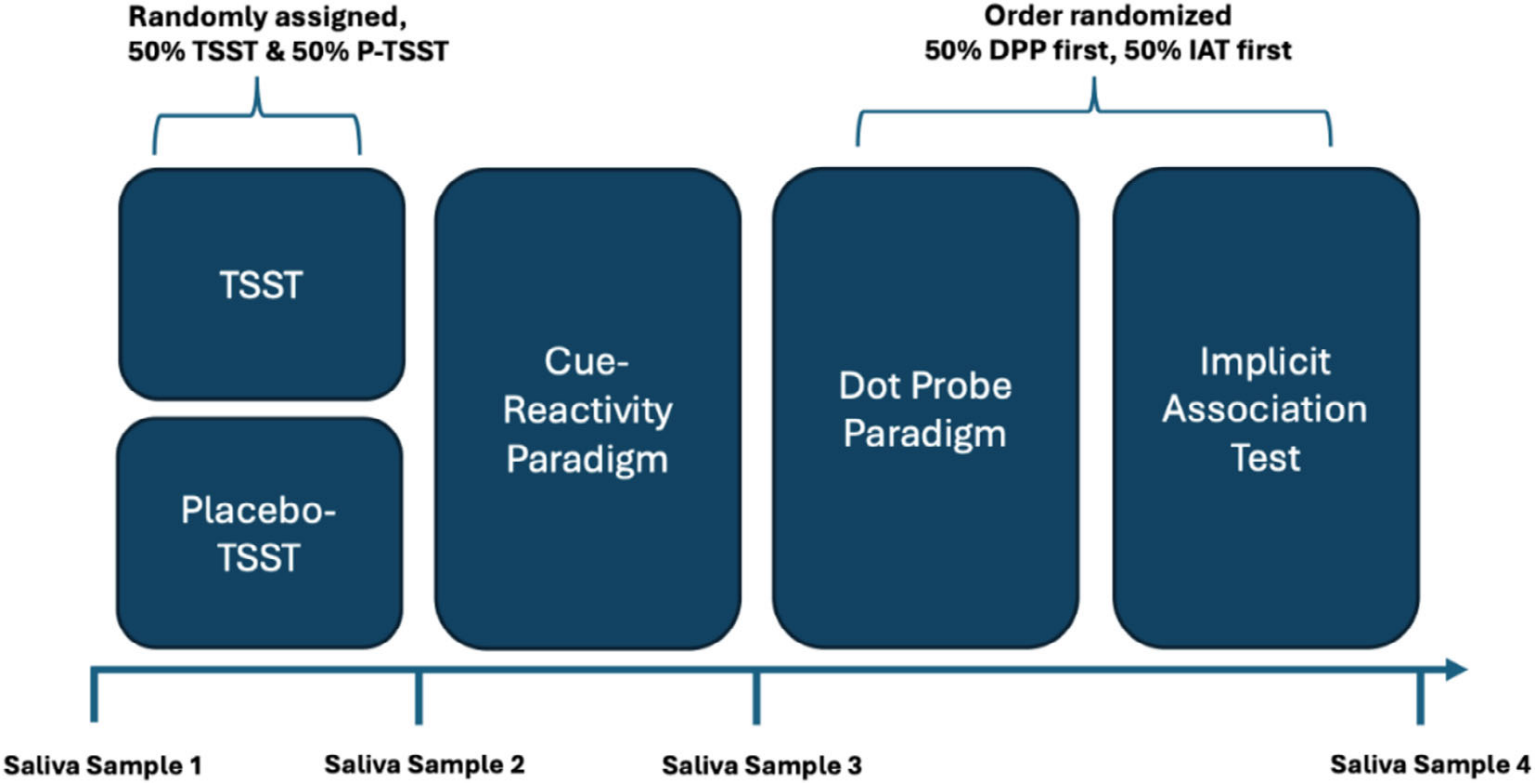
Mean salivary cortisol [2(a)] responses of participants with pathological social network use (p‐SNU group) and the control group (CG) exposed to either the Trier social stress test (TSST) or the placebo‐TSST (p‐TSST). Error bars represent standard errors of the mean. ***P < 0.001, *P < 0.05 TSST compared to the p‐TSST. See Table S1(a) for all mean (SD) and 95% CI values. *Note:* Illustration of the study design including the time course. DPP, Dote Probe Paradigm; IAT, Implicit Association Test; P‐TSST, Placebo‐TSST.

The procedure applied is part of a multi‐center German Research Foundation‐funded addiction research unit (FOR2974) (doi.org/10.17605/OSF.IO/N5CD7) on affective and cognitive mechanisms of specific internet‐use disorders (ACSID). The sub‐project's specific procedure and hypotheses of the project are pre‐registered at the Open Science Framework (doi.org/10.17605/OSF.IO/EHQ98). After the participants completed the core battery of the FOR2974 [43], the project‐specific part was carried out. This included a random assignment to the Trier social stress test (TSST, see description below) or placebo‐TSST. Afterward, the Cue‐Reactivity Paradigm (CRP) as well as the measurements for implicit cognitions, namely the Implicit Association Test (IAT) and Dot Probe Paradigm (DPP) followed. To ensure a balanced distribution, 50% of the participants first conducted the DPP and 50% the IAT. Saliva samples for the determination of cortisol (and stress responses) were collected before the TSST or placebo‐TSST (t0), after the TSST or placebo‐TSST (t1:25 minutes after t0), after the CRP (t2:40 minutes after t0), and after the paradigms for measuring implicit cognitions (t3:60 minutes after t0). To minimize the effects of the natural circadian rhythm, the TSST/placebo‐TSST and project‐specific behavioral tasks took place in the afternoon [44] (see Figure 1 for the visualization of the study procedure).

### Participants

Recruitment took place from December 2021 to June 2023 at the University of Duisburg‐Essen and the Hannover Medical School through mailing lists, SN or word‐of‐mouth recommendations. For the exclusion criteria, see Supplemental material A1. Participants had to be a clinically relevant case for p‐SNU (p‐SNU group) or classified as individual with non‐problematic use indicated as control participants (CG group) to participate in the study. This was determined with the administration of a standardized diagnostic interview to assess specific internet use disorders. In a first step, a standardized diagnostic telephone interview (AICA‐C9) was conducted with all participants based on the Diagnostic and Statistical Manual of Mental Disorders (DSM)‐5 criteria for gaming disorder, adapted for p‐SNU. In clinically relevant cases for confirming the classification, the AICA‐SCI:IUD was additionally implemented, which is a slightly adapted version of the AICA‐SKI:IBS [45]. If five or more symptoms of p‐SNU were present, participants were classified as individuals with p‐SNU. Only a maximum of one criterion was allowed for participants to be assigned to the control group. Because we used on‐line buying shopping‐cues as control stimuli, it was ensured that within the current sample, individuals only showed non‐problematic or at most risky on‐line buying‐shopping behaviors (fulfilment of max. 4/9 criteria from the structured interview). Higher scores (>4 criteria fulfilled) were an exclusion criterion for both the p‐SNU and the CG. Interviews were conducted by trained PhD students (M.J., K.T., A.K.) who were regularly supervised (E.W., A.M.). Approval of the entire study protocol was obtained from the local ethics committee of the University of Duisburg‐Essen (ID: 1911APBM0457), and the Hannover Medical School (8767_BO_S_2019), which was conducted in a similar manner at the respective sites of this multi‐center research unit. All participants gave voluntary informed consent. Because of the higher presumed prevalence in women with p‐SNU [46] and considering the overall research question of this research project, this study collected data from a total of 135 female participants age 18 to 41 years [mean (M) = 24.38, SD = 4.46]. Using the AICA‐C9 and AICA‐SCI:IUD as diagnostic procedure, 64 of the participants were assigned to the CG group, whereas 71 participants were allocated to the p‐SNU group. The groups did not differ regarding age or other socio‐demographic variables; only the daily use time was significantly higher in the p‐SNU group (see Table 1).

**TABLE 1:** Descriptive statistics of the p‐SNU group and the control group, as well as inference statistical values of the group comparisons.

Group	p‐SNU (*n* = 71)		Control group (*n* = 64)		Group comparison		
	M (SD/%)	Range	M (SD)	Range	*t*_131_	*P*	│d│
Age (in y)	24.44 (3.92)	19–37	24.31 (5.02)	18–41	−0.161	0.872	0.028
Daily use time (in minutes)	234.51 (117.61)	77.50–750.00	177.69 (105.73)	30.00 – 465.00	−3.126	0.002	0.542
Qualification for university entrance					χ^2^		ϕ
Yes	66 (93.0%)		58 (90.6%)		4.31	0.366	0.179
No	6 (7.0%)		6 (9.4%)				
In school, vocational training, studying							
Yes	56 (78.8%)		49 (76.6%)		7.60	0.369	0.237
No	15 (21.2%)		15 (23.4%)				
Living in committed partnership							
Yes	46 (64.8)		32 (50.0)		3.02	0.082	−0.250
No	25 (35.2)		32 (50.0)				

Abbreviation: p‐SNU, problematic social network use; M, mean value; SD, standard deviation.

### Measures

#### Stress induction

*TSST and placebo‐TSST*. The TSST [47] is a standardized procedure to induce acute psychosocial stress. Participants were instructed, after a short preparation time (5 minutes), to perform a free speech and a mental arithmetic task in front of a panel consisting of two people (a woman and a man). The committee should behave in a detached and reserved manner without showing emotional expressions. For the present study, the male part was leading the free speech part while the female part of the jury was responsible for the arithmetic task. The placebo‐TSST (p‐TSST) was used as a control condition without stress induction [48]. The socio‐evaluative components of the TSST were missing here, as the participants gave a presentation to themselves alone in the test room and the mental arithmetic task consisted of counting in steps of 15.

*Saliva stress measures*. A total of four saliva samples were used to measure the physiological effects of stress. Because stress induction is associated with a delayed salivary cortisol (sCort) response of the HPA axis and shows rapid effects on salivary alpha amylase (sAA) reactivity of the sympathetic nervous system [49, 50], both values were used to identify responders for stress induction. sCort responders were identified by an increase of ≥1.5 nmol/L, whereas values of >10% were required for sAA [51]. The biomarkers were evaluated at the Department of Cognitive Psychology, Institute of Cognitive Neuroscience at Ruhr University Bochum.

### Experimental paradigms

For all the following paradigms, presentation software (Neurobehavioral Systems, Berkley, CA) was used to present the stimuli and to record the behavioral responses. Additionally, for the IAT and DPP, a response pad (Cedrus Response Pad RB844, San Pedro, CA) was used on which participants had to react with two colored buttons (following the respective instruction). For all paradigms, SN‐related stimuli were used as addiction‐related target stimuli to examine the target behavior and buying‐shopping‐related stimuli as non‐addiction‐related, control stimuli (= control condition). Because of the emerging similarities across p‐SNU and p‐BSh and the overall research aim of the project, this study was implemented for either p‐SNU as target behavior or p‐BSh as target behavior with specific control groups for each on‐line behavior [52]. The present results are solely focused on the p‐SNU group as target.

#### Cue‐Reactivity Paradigm

The CRP consists of 20 SN‐related stimuli that represent login pages of various typical, frequently used SN and instant messaging services. The 20 control stimuli show login pages of the most‐used German on‐line buying‐shopping sites. Participants were asked about their preferred terminal device to use SN (smartphone, tablet, laptop or computer), which was then used as a frame for the login‐pages. All stimuli were presented in blocks of 10 stimuli (pseudorandomized, balanced order: target vs. control stimuli) (for a more detailed description see Diers *et al*. [21]). Each stimulus had to be evaluated with respect to arousal, urge and valence to use the application on a 5‐point Likert scale ranging from 1 (=no urge) to 5 (=very strong urge). For the analyses, the mean arousal, urge and valence toward the SN stimuli were used as measurements of cue‐reactivity and craving.

#### Implicit Association Test

A modified version of the IAT [53, 54] was used to assess implicit (automatic) associations toward SN‐related stimuli. During the IAT, visual stimuli had to be categorized as fast as possible according to target (‘SN‐related stimuli’ vs. ‘control stimuli’) and attribute concepts (‘positive’ vs. ‘negative’) by using two buttons on a response pad. A detailed description can be found in Supplemental material A2. As dependent variable, the D2D score was used. The D2D value was computed as the difference in reaction times between the incongruent pairings and the congruent pairings divided by their overall standard deviation [55]. Higher D2D scores indicate stronger positive implicit associations with SN‐related stimuli.

#### Dot Probe Paradigm

A visual DPP that has been applied in a previous study on buying‐shopping disorder [56] was used to measure participants' attentional bias toward SN‐related stimuli. In total, 20 SN‐related stimuli and 20 control stimuli were used. An attentional bias score was calculated for each participant by subtracting the mean latency (ms) to respond to a probe replacing an addiction‐related stimulus (congruent trial) from the mean latency to respond to a dot replacing a control stimulus (incongruent trials). Further descriptions can be found in Supplemental material A3. Positive values for the attentional bias score suggest an orientation toward the SN‐related visual cues.

#### Statistical analysis

Power analysis with GPower (version 3.1.9.2) showed that for testing our main hypotheses a total sample size of *n* = 128 is required to detect a medium‐sized effect of stress induction compared to the control condition with a power of 0.80. Four individuals were excluded because of missing values in the biological markers and paradigms; only complete data sets were included in the analysis. Because we recruited beyond the targeted sample size, a sufficiently large data set was still available for the analysis. Between group comparisons (e.g. demographic data) were analyzed using independent or paired *t* tests or χ^2^ tests. Data with regard to the effect of the stress induction (TSST) compared to the non‐stress condition (p‐TSST) on implicit cognitions (measured with DPP and IAT)were analyzed using repeated measures analysis of variance (ANOVA) with the within‐subjects factor ‘stimulus category’ (SN‐related vs. control stimuli) and the two between subjects factors ‘group’ (p‐SNU vs. control group) and ‘stress condition’ (TSST vs. placebo‐TSST). Tests for variable distribution, potential outliers and normality of the residuals were conducted. If the normality assumptions were violated, we applied robust standard errors to ensure more accurate *P*‐values and CI. Greenhouse‐Geisser correction was carried out in the case of sphericity violation. For the interaction effect with craving, interactions between stress condition and urge, arousal and valence (from the CRP) for SN‐related stimuli on task performance in the DPP or the IAT were analyzed with hierarchical moderated regressions. The significance level was defined as *P* < 0.05; Cohen's *d* (*t* tests), φ coefficients (χ^2^ tests), partial ηp^2^ (ANOVAs) or *R* ^*2*^ (regressions) are specified for the effect sizes. All analyses were performed with IBM SPSS Statistics Version 29.0.1.0 (IBM, Armonk, NY).

## RESULTS

### Manipulation check—TSST effects/biomarker results

**FIGURE 2:**
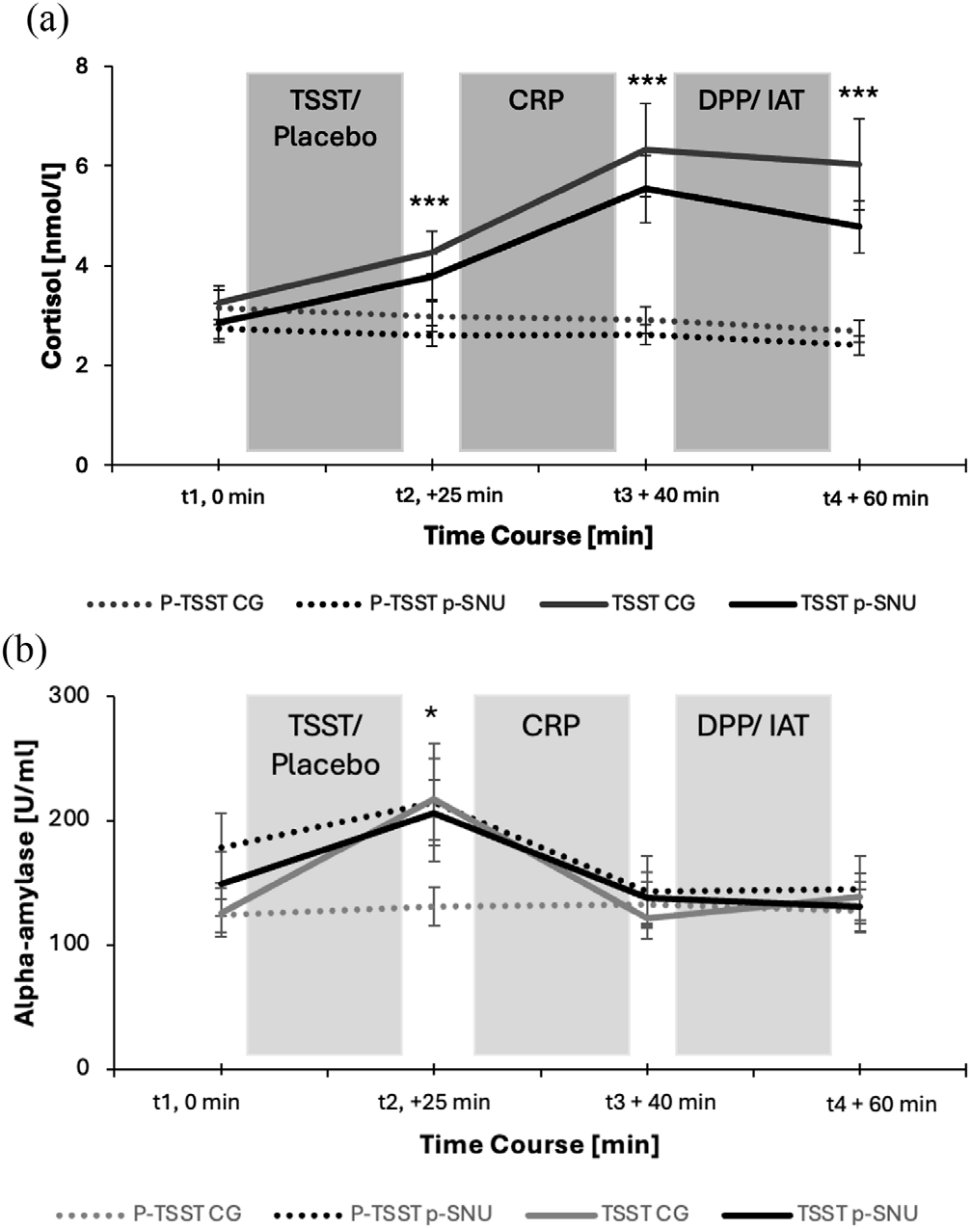
Mean salivary cortisol (a) and α‐amylase (b) responses of participants with problematic social network use (p‐SNU group) and the control group (CG) exposed to either the Trier Social Stress Test (TSST) or the placebo‐TSST (p‐TSST). Error bars represent standard errors of the mean. ***P < 0.001, *P < 0.05 TSST compared to the p‐TSST. See Tables S1 and S2(a) for all mean (SD) and 95% CI values. CRP, Cue‐Reactivity Paradigm; DPP, Dot Probe Paradigm; IAT, Implicit Association Test.

Regarding the relevance of acute stress, we used the proposed value of Miller *et al*. [51] with a sCort increase of >1.5 nmol/L to identify responders of stress induction. In total, 32 participants were identified as sCort responder (16 p‐SNU and 16 control participants). Thirty‐one of them were in the TSST condition, and only one responder was in the p‐TSST condition. All participants remained in the analysis to test the hypotheses. Visualizing the stress responses, the sCort peak was reached at t3 (see Figure 2a).

All mean scores and confidence intervals of the stress responses using sCort are shown in Table S1. The statistical analysis showed a significant main effect of time, *F*(1.37, 133.15) = 17.68, *P* < 0.001, η_p_^2^ = 0.12 and a time by stress condition interaction at t2, t3 and t4, *F*(1.37, 200.60) = 26.63, *P* < 0.001, η_p_^2^ = 0.17. No significant time by group interaction, *F*(1.37, 200.60) = 0.34, *P* = 0.631, η_p_^2^ = 0.01 or time by group by stress condition interaction emerged, *F*(1.37, 200.60) = 0.83, *P* = 0.397, η_p_^2^ = 0.01.

All mean scores and confidence intervals of the sAA are shown in Table S2. The sAA peak was reached at t2 (Figure 2b). There was a significant main effect of time, *F*(1.37, 133.15) = 21.06, *P* < 0.001, η_p_^2^ = 0.14, as well as a significant time by stress condition interaction at t2, *F*(1.37, 200.60) = 3.84, *P* < 0.05, η_p_^2^ = 0.03, and of time by group by stress condition, *F*(1.37, 200.60) = 3.07, *P* < 0.05, η_p_^2^ = 0.02. Regarding time by group, there was no significant effect, *F*(1.37, 200.60) = 2.07, *P* = 0.120, η_p_^2^ = 0.02.

### Manipulation check—Cue‐Reactivity Paradigm

**FIGURE 3:**
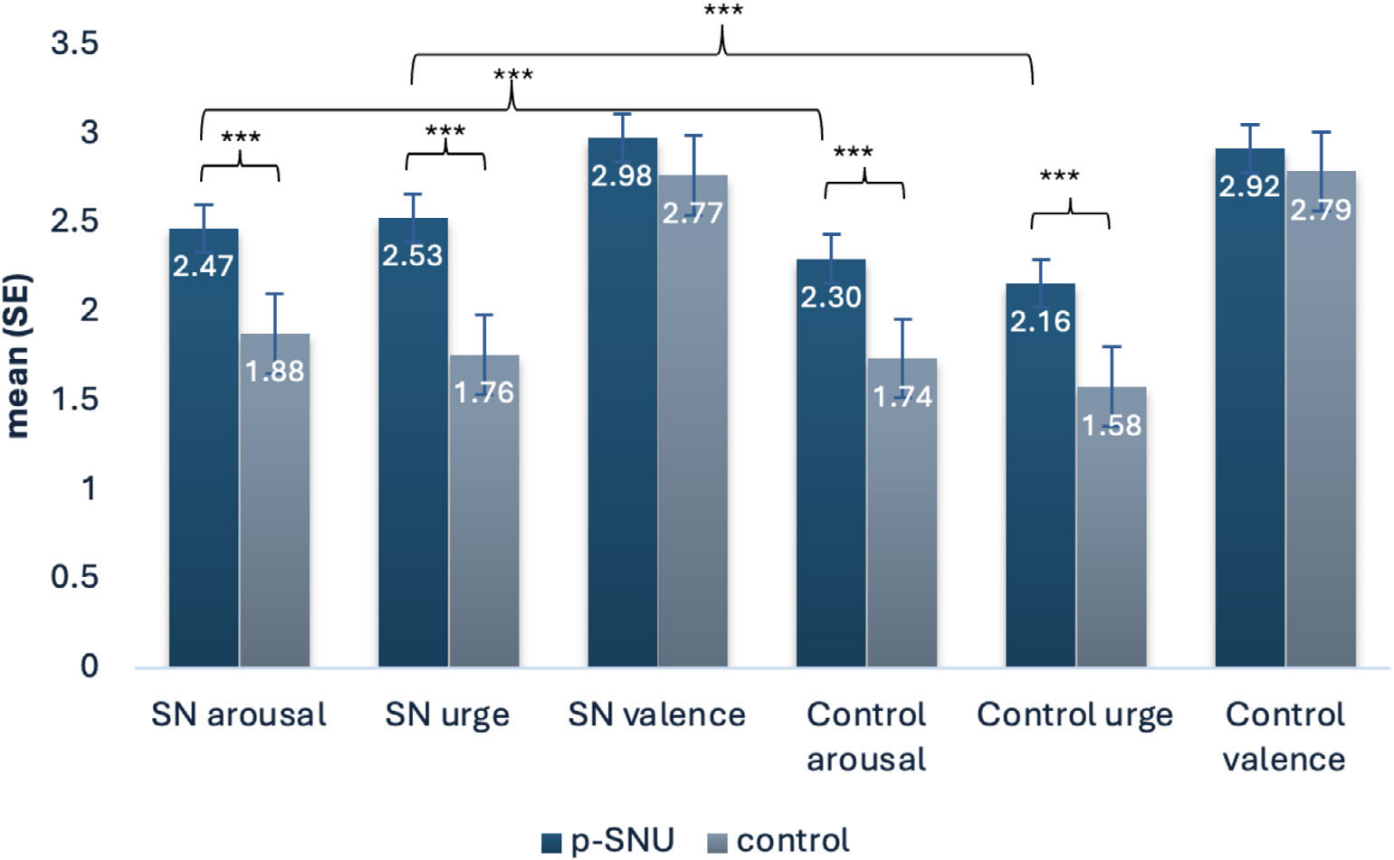
Overall subjective ratings of social network (SN)‐related cues (pictures of login pages of social networks) and control cues (pictures of login pages of shopping websites) for individuals with problematic social network use (p‐SNU) and the control group (CG). Error bars represent standard errors of the mean. ***P < 0.001. All mean scores, confidence intervals, and the group comparisons are shown in Table S3.

The manipulation test, to determine whether the SN stimuli were generally rated higher than the control stimuli, reveals that the p‐SNU group showed higher ratings to the SN‐stimuli in subjective arousal and subjective urge (Figure 3). Regarding the control stimuli, the p‐SNU group showed higher ratings in subjective arousal and subjective urge than the CG, but no higher subjective valence.

Within the group of individuals with p‐SNU, they had a higher subjective arousal *t*_70_ = 2.55, *P* = 0.013, │*d*│ = 0.30 and subjective urge for SN‐related stimuli *t*_70_ = 5.40, *P* < 0.001, │*d*│ = 0.58 than for control stimuli, but no higher subjective valence, *t*_70_ = 1.34, *P* = 0.185 │*d*│ = 0.45. The CG rated the SN‐related stimuli with higher subjective arousal *t*_63_ = 2.76, *P* = 0.007, │*d*│ = 0.38 and subjective urge, *t*_63_ = 3.45, *P* < 0.001, │*d*│ = 0.41 than the control stimuli, but there was no significant difference in subjective valence *t*_63_ = − 0.55, *P* = 0.583 │*d*│ = 0.27.

### Hypothesis 1

#### Effects of acute stress on cue‐reactivity and craving

The p‐SNU group had stronger subjective arousal and subjective urge but no higher subjective valence for SN‐related stimuli than the CG (Table 2). The main effect of stress was significant and indicates that the TSST group had a higher subjective urge for SN‐related stimuli than the placebo‐TSST group, *F*(1, 131) = 6.820, *P* = 0.01, ηp^2^ = 0.029. There was no significant stress effect of subjective arousal between the TSST and p‐TSST, *F*(1, 131) = 1.842, *P* = 0.177, ηp^2^ = 0.014. Additionally, subjective valence was not higher in the TSST group versus the p‐TSST group, *F*(1, 131) = 0.722, *P* = 0.397, ηp^2^ = 0.005 (Table 2).

**TABLE 2:** CRP values of SN‐stimuli and group differences between p‐SNU and the control group and TSST versus p‐TSST.

Group	p‐SNU (*n* = 71)		Control group (*n* = 64)		Group comparison		
	M (SD)	95% CI	M (SD)	95% CI	*t*_131_	*P*	│d│
Arousal	2.47 (0.81)	2.28–2.67	1.88 (0.79)	1.68–2.08	−4.32	<0.001	0.80
Urge	2.53 (0.91)	2.32–2.74	1.76 (0.80)	1.57–1.96	−5.18	<0.001	0.86
Valence	2.98 (0.59)	2.85–3.12	2.77 (0.74)	2.59–2.96	−1.81	0.072	0.31
TSST condition	TSST (n = 64)		p‐TSST (n = 71)		*F*(131)		ηp^2^
Arousal	2.27 (0.83)	2.08–2.49	2.11 (0.86)	1.91–2.33	1.842	0.177	0.014
Urge	2.26 (0.92)	2.04–2.49	2.08 (0.96)	1.86–2.33	6.820	0.01	0.029
Valence	2.93 (0.64)	2.77–3.09	2.84 (0.70)	2.68–3.00	0.722	0.397	0.005

Abbreviations: CRP, Cue‐Reactivity Paradigm; Arousal, CRP arousal; Urge, CRP urge; Valence, CRP valence; p‐TSST, placebo‐TSST; p‐SNU, problematic social network use; SN, social network; TSST, Trier Social Stress Test.

The interaction effect of the group by stress proves to be non‐significant for subjective arousal, *F*(1, 131) = 0.001, *P* = 0.994, ηp^2^ = 0.001, CI = 0.00–0.01; subjective urge, *F*(1, 131) = 0.592, *P* = 0.442, ηp^2^ = 0.005, CI = 0.00–0.05; and subjective valence, *F*(1, 131) = 0.022, *P* = 0.882, ηp^2^ = 0.001, CI = 0.00–0.01.

### Hypothesis 2

#### Effects of acute stress on implicit cognitions

Analyzing the effect of acute stress on implicit cognitions, the p‐SNU and control group differed neither regarding their expression of attentional bias nor regarding the congruent or incongruent pairing. Additionally, the p‐SNU group did not show higher implicit associations (Table 3).

**TABLE 3:** Task performance in the DPP and IAT of individuals with p‐SNU compared to the control group and TSST versus p‐TSST group.

Group	p‐SNU (*n* = 71)	Control group (*n* = 64)	ANOVA		
			*F*(1, 131)	*P* [95% CI]	η_p_^2^
Attentional bias score, M (SD)	−0.17 (17.37)	2.60 (17.48)	0.83	0.363 [0.00–0.06]	<0.01
Reaction time in congruent trials^a^ [ms], M (SD)	359.67 (59.87)	347.95 (41.98)	1.64	0.202 [0.00–0.07]	<0.01
Reaction time in incongruent trials^b^ [ms], M (SD)	359.50 (54.33)	350.56 (41.07)	1.10	0.294 [0.00–0.06]	<0.01
D_2D_, M (SD)	0.49 (0.39)	0.43 (0.05)	0.01	0.963 [0.00–0.02]	<0.01
TSST condition	TSST (*n* = 64)	p‐TSST (*n* = 71)			
Attentional bias score, M (SD)	1.75 (18.88)	−1.10 (18.86)	0.45	0.506 [0.00–0.05]	<0.01
Reaction time in congruent trials^a^ [ms], M (SD)	356.65 (59.98)	348.56 (41.92)	0.05	0.819 [0.00–0.03]	<0.01
Reaction time in incongruent trials^b^ [ms], M (SD)	358.39 (57.71)	347.46 (39.79)	0.23	0.630 [0.00–0.04]	<0.01
D_2D_, M (SD)	0.45 (0.42)	0.51 (0.45)	0.01	0.951 [0.00–0.02]	<0.01

Abbreviations: DPP, Dot‐Probe Paradigm; IAT, Implicit Association Test; M, mean; p‐TSST, placebo‐TSST; p‐SNU, problematic social network use; TSST, Trier Social Stress Test.

^a^ DPP following SN‐related stimuli.

^b^ DPP following control stimuli.

No significant group by stress interactions were found in the attentional bias score, *F*(1, 131) = 3.303, *P* = 0.071, η_p_^2^ = 0.025, CI = 0.00–0.09, reaction time in congruent trials, *F*(1, 131) = 0.134, *P* = 0.715, η_p_^2^ = 0.01, CI = 0.00–0.04, or reaction time in incongruent trials, *F*(1, 131) = 0.062, *P* = 0.803, η_p_^2^ = 0.0, CI = 0.00–0.03, or D2D score, *F*(1, 131) = 0.521, *P* = 0.472, η_p_^2^ = 0.01, CI = 0.00–0.03. We additionally calculated separate *t* tests for independent groups. Although no significant results were found regarding implicit associations, results showed that individuals in the control group exhibited a higher attentional bias toward SN‐related stimuli than the p‐SNU group in the p‐TSST condition, *t*_67_ = 2.136, *P* = 0.036, │*d*│ = 0.52, CI = 0.53–15.82, indicating a middle size effect. No other significant differences were found.

### Hypothesis 3

#### Interaction with subjective arousal, urge and valence

Investigating the interaction of stress condition and subjective arousal, urge and valence on attentional bias and implicit association, moderated regression analyses showed neither significant effects of stress response or subjective arousal, urge or valence responses, nor moderated effects by arousal, urge and valence responses of acute stress on implicit cognitions within individuals with p‐SNU (Table 4).

**TABLE 4:** Interactions of urge, arousal and valence and TSST condition on implicit cognitions, *n* = 70.

	DPP (attentional bias score)						IAT (D_2D_)					
	B	SE	β	*t*	*P* [95% CI]	*R* ^*2*^	B	SE	β	*t*	*P* [95% CI]	*R* ^*2*^
TSST condition	3.49	4.15	0.10	0.84	0.40 [−4.79 to 11.77]		−0.05	0.09	−0.06	−0.50	0.61 [−0.24 to 0.15]	
Urge	−4.12	2.23	−0.22	−1.84	0.07 [−8.44 to 0.44]		−0.03	0.05	0.07	−0.54	0.59 [−0.13 to 0.08]	
Arousal	4.37	4.14	0.13	1.06	0.29 [−9.39 to 0.67]		−0.04	0.09	−0.05	−0.41	0.68 [−0.15 to 0.08]	
Valence	3.97	4.14	0.11	0.96	0.34 [−12.53 to 1.34]		−0.05	0.09	−0.06	−0.48	0.63 [−0.20 to 0.12]	
TSST condition × urge	3.67	4.49	0.10	0.82	0.42 [−5.30 to 12.64]	0.07	‐0.02	0.10	−0.03	−0.22	0.82 [−0.23 to 0.18]	0.01
TSST condition × arousal	1.66	5.12	0.04	0.32	0.75 [−8.56 to 11.89]	0.06	0.01	−12	0.01	0.02	0.98 [−0.24 to 0.24]	0.01
TSST condition × valence	−1.49	7.08	−0.03	−0.21	0.83 [−15.63 to 12.65]	0.05	−0.26	0.16	−0.19	−1.60	0.11 [−58 to 0.06]	0.04

*Note*: TSST condition: TSST vs. placebo‐TSST.

Abbreviations: TSST, Trier Social Stress Test; Urge, CRP urge; Arousal, CRP arousal; Valence, CRP valence; DPP, Dot‐Probe Paradigm; IAT, Implicit Association Test.

### *Post hoc* analysis

Because of the insignificant moderation effect and after re‐examining the causal link regarding our study setting and the underlying literature [39, 40], it can also be assumed that the subjective urge is possibly triggered by the acute stress but does not necessarily affect the implicit cognition in the interaction but rather mediates the effect of stress on implicit cognitions. For this reason, a mediation analysis (not pre‐registered) may additionally be conducted to examine the possible mediating effect of subjective urge on the relationship between stress and the expression of implicit cognitions in the p‐SNU group. However, the pre‐requisites regarding the bivariate correlations are not met, and the mediation analysis cannot be carried out (see Table S4 in the Supplemental material). The stress condition is neither correlated with the measurements of attentional bias (*r* = 0.076, *P* = 0.521) nor with implicit associations (*r* = −0.057, *P* = 0.634).

## DISCUSSION

The aim of this study was to investigate the effects of acute psychosocial stress on cue‐induced craving and implicit cognitions in p‐SNU and to compare these effects with individuals not suffering from p‐SNU. We found that acute stress did not lead to higher cue‐induced craving reactions measured as subjective urge, arousal, valence, and implicit cognitions in women with p‐SNU compared to the control group. This is not consistent with the assumptions of theoretical models of stress [7, 57, 58], the I‐PACE model [6], and previous empirical research (e.g. Schröder and Mühlberger [41]). Nevertheless, individuals with p‐SNU were disposed to a higher subjective urge and arousal for SN‐related stimuli than non‐problematic users, whereas stress leads to a higher subjective urge for SN use in both groups. Contrary to our assumptions, in the placebo stress condition, the control group showed a higher attentional bias toward SN‐related stimuli than the p‐SNU group. Inconclusive findings were obtained regarding the effects of stress on implicit cognitions or an interaction between stress and subjective urge on implicit cognitions.

Discussing the results of the physiological biomarkers, they revealed increasing sCort and sAA levels in the stress condition and the same number of responders in the p‐SNU as in the control group. It indicates moderate stress induction in half of the sample [49]. The discrepancy in the expected response rate could be because of the individual variability of HPA axis reactivity and potential contextual factors associated with the administration of the TSST. Cortisol levels show that in both conditions, individuals start with similar baseline levels and increase in both groups because of stress induction. In contrast to previous study, results with attenuated HPA stress response toward acute psychosocial stress in individuals with substance or internet use disorders compared to healthy control participants [15, 16, 59], the adaptation of the HPA axis within the 1‐hour period examined in this study does not appear to differ between individuals with and without p‐SNU. This may suggest similar long‐term adaptations and regulatory mechanisms of stress responses, which cannot be further interpreted based on the present study's findings. In contrast, the p‐SNU group significantly has higher levels of sAA from the outset (i.e. the rapid, immediate response of the sympathetic nervous system), which also increases independently of the stressor using TSST or p‐TSST. This means that individuals with p‐SNU have a higher fast stress response in unforeseeable situations even without the combination of demanding cognitive tasks and social evaluation. At the same time, the observed cortisol increase in the p‐SNU group might be lower than expected because this group may no longer show optimal adaptation to the stress situation.

The control group reacted to acute stress in a similar way to the p‐SNU group, and it is not yet clear to what extent SN can be used adaptively to reduce stress even in the case of unproblematic use [60]. Furthermore, our results support the assumption of sensitized reward processing in addictive behaviors [61, 62]. They are in line with recent study results on successful cue‐induced craving with distal cues in other on‐line behavioral addictions [21]. According to this, even log‐in pages without displaying the actual SN content seem to be sufficient to trigger an increased urge in individuals with p‐SNU. However, as most users remain logged in on their devices and rarely encounter these pages in everyday use, the ecological validity of this trigger should be critically considered even if a first study illustrates the effect of log‐in pages inducing cue reactivity and craving in on‐line gaming [21]. Future research may benefit from using more naturalistic cues, such as notifications or personalized content previews, to better reflect real‐world conditions. At the same time, the results demonstrate that the cues trigger higher urge and arousal ratings in individuals with p‐SNU, which probably indicates that the cues are valid and individuals with p‐SNU may have developed more generalized cue reactivity and craving, even to not‐individualized distal cues such as log‐in pages/symbols.

As stress increases craving in both groups, it could be hypothesized that the use of SN serves different functions. Although SN may be used by non‐problematic users as a temporary relief without negative consequences, it could lead to more compulsive use and negative consequences in individuals with p‐SNU [18]. This is supported by a recent study by Zhao *et al*. [63], showing that stress triggers the desire to use SN regardless of the severity of symptoms and that there is no clear evidence for the difference in the active and passive intention to use SN. In this controlled study setting, stress appeared to heighten the urge to use SN, a pattern that may also extend to everyday situations, possibly even with unproblematic use.

In addition, the experience of craving may also be associated with changes in biological stress responses and may represent a state of experienced (emotional) stress even in individuals without mental disorders [64]. If SN are used as adaptive coping strategies, this may also lead to automatic tendencies via habituation and promote an effect in implicit cognitions without any noticeable negative consequences for individuals with healthy, recreational use [18]. This could explain the more pronounced attentional bias in the non‐problematic users, which is only slightly but significantly higher than in individuals with p‐SNU. Compared to other on‐line behavioral addictions (e.g. shopping [56]), the functional use of SN may lead to increased attention. One possible reason is that even individuals with non‐problematic usage of SN use them for several hours a day and habitual behavior patterns can take place even without higher symptom severity. This unique feature of SN use could set it apart from other on‐line behaviors or disordered use. The higher attentional bias disappeared when stress was triggered, which may indicate that acute stress tends to increase affective components such as acute craving and limit cognitive mechanisms in non‐problematic use. According to current research, it is questionable whether implicit positive associations and increased attention are present at all in problematic stages of SN use [38]. Our results partly suggest that they tend to play a greater role in non‐problematic use or in earlier stages of the development of p‐SNU, where reward‐oriented and gratifying consumption motives are predominant. At later stages, these motives may be replaced by compensation‐oriented mechanisms or compulsive behavioral tendencies [6]. The findings of Ihssen and Wadsley [65] also support this, showing that the ‘wanting’ to use SN significantly predicted the severity of p‐SNU symptoms, but not the ‘liking’ part, which could also explain why the positive attributes measured with the IAT do not seem to play a role in p‐SNU.

Wrapping up, although our results do not establish causality, they suggest that negative emotional states (current mood) have only a weak association with affective responses to cues. Snapshots of negative emotional states such as stress should, therefore, not be underestimated nor should the importance of cognitive mechanisms, which could be assumed to act as a mediator of avoidance expectancies and p‐SNU [63]. The constant exposure to external stimuli of SN and the ubiquitous, easy accessibility of SN enables quick, short‐term reward moments that can override compensatory stress management. Based on these findings, it is necessary to consider a holistic interplay of different cognitive mechanisms.

### Limitations and future directions

It cannot be ruled out that the peak of the stress response was reached during the processing of the paradigms, making it difficult to draw a holistic conclusion about the physiological stress response based on the timing of the saliva samples. Furthermore, the paradigms contained control stimuli representing log‐in pages for on‐line shopping or icons, which could also influence possible effects. Dot‐Probe or dual tasks also have been criticized in the literature for their lack of reliability because of their reliance on them (e.g. Jones *et al*. and Ataya *et al*. [66, 67]), and although experimental measurements might provide a more reliable alternative, they come with their own challenges, such as increased complexity and the potential for higher noise in the data, which constitutes a limitation of the study. We suggest that future research needs to address these challenges more systematically to provide the best opportunity to measure attentional biases. One important aspect to consider when interpreting our results is the statistical approach used to model stress reactivity. Given the observed variability in cortisol and α‐amylase reactivity, alternative approaches such as linear mixed models may allow for a more nuanced understanding of these individual differences [68]. Future studies could benefit from hierarchical models or robust statistical approaches [69] to allow a more flexible examination of the interindividual variability of stress responses and their associations with cognitive measures.

Because most control participants also met the criterion of impaired control in the structured interview, reduced inhibitory control could also significantly influence the effects of psychosocial stress and obscure unconscious cognitive mechanisms. In addition, prolonged exposure to chronic stress can lead to reversible changes in brain regions that can also alter affective and cognitive mechanisms [70]. It is, therefore, important to take a closer look at the extent to which actual response inhibition in our sample may have influenced the mechanisms investigated here. The generalizability of the results is limited to a sample of German women in their mid‐twenties with a high level of education. Future studies should investigate how everyday stressful situations contribute to an increased desire to use SN and trigger its use as an automatic default behavior to compensate for the resulting negative emotional state. The experimental TSST condition may cause individuals to behave differently because of social pressure compared to their reactions to acute stress in their usual environment. To explore this further, ecological snapshots could be used to examine whether SN serve as coping or emotion regulation strategies in these moments, as demonstrated in a previous study by Fatseas *et al*. [64]. Additionally, it is also crucial to investigate the underlying motives for SN use in stressful situations. Because existing research in this area is still very limited, future studies should determine whether the intention to use SN in such moments is primarily driven by actively seeking social support or whether they function more as a blunt distraction tool, with passive use being the main focus [63].

## CONCLUSIONS

These study results indicate that acute stress is an influencing factor for cue‐induced craving in both individuals with non‐problematic SN use and individuals with p‐SNU. Although the affective mechanisms are more pronounced in p‐SNU, cognitive attentional processes also appear to take place in individuals with non‐problematic use. These are not reflected in positive implicit associations, but are distinct from those prevailing in p‐SNU. In the initial phase, SN appear to attract the attention of individuals with non‐problematic use more strongly, which could influence the decision to use them, while in later phases this could be achieved more through a higher urge and habitual behavior patterns. Against the background of the uniqueness of the SN usage behavior, but also the similarities to other on‐line behavioral addictions, this study makes an important contribution to the current state of research on p‐SNU, which underlines the relevance of further research.

## AUTHOR CONTRIBUTION

**Annica Kessling:** Data curation; formal analysis; investigation; visualization; writing—original draft; writing—review and editing. **Astrid Müller:** Conceptualization; formal analysis; funding acquisition; methodology; project administration; supervision; visualization; writing—original draft, writing—review and editing. **Oliver T. Wolf:** Conceptualization; funding acquisition; methodology; project administration; writing—review and editing. **Christian J. Merz:** Data curation; formal analysis; writing—review and editing. **Matthias Brand:** Conceptualization; funding acquisition; writing—review and editing. **Elisa Wegmann:** Conceptualization; formal analysis; funding acquisition; methodology; project administration; supervision; writing—review and editing.

## DECLARATION OF INTERESTS

The authors report no financial or other conflict of interest relevant to the subject of this presentation.

## CLINICAL TRIAL REGISTRATION

The sub‐project's specific procedure and hypotheses of the project are pre‐registered at the Open Science Framework (doi.org/10.17605/OSF.IO/EHQ98).

## Supporting information


**Table S1.** Salivary cortisol (sAA) values of Figure 2a within the time course t1‐t4. Note. P‐TSST: Placebo‐TSST. T1 = baseline, t2 = +25 min, t3 = +40 min, t4 = +60 min.
**Table S2.** Alpha‐Amylase values of Figure 2b within the time course t1‐t4. Note. P‐TSST: Placebo‐TSST. T1 = baseline, t2 = +25 min, t3 = +40 min, t4 = +60 min.
**Table S3.** Mean values of control stimuli of the Cue‐Reactivity Paradigm. Note. Control stimuli: online buying‐shopping stimuli.
**Table S4.** Correlational Analysis of stress condition, implicit cognition measures and Craving response by the Cue‐Reactivity Paradigm. *n* = 70. Note. TSST condition: TSST vs. p‐TSST, Subjective urge: CRP urge; Subjective arousal: CRP arousal, Subjective valence: CRP valence, **P < 0.01.

## Data Availability

In the interests of open science practice, access to the research group's data will be published as soon as the data collection of the entire research group has been completed. The data that support the findings of this study are available on request from the corresponding author, [AK].
