# Supplementary material for: Effects of acute psychosocial stress on cue‐reactivity, attentional bias and implicit associations in women with problematic social network use: An experimental study
Source: Addiction. 2025 Jun 13;120(10):2067–79. doi: 10.1111/add.70099 (PMC12426358; doi:10.1111/add.70099)
Supplement: Supplementary file 1 — Table S1. Salivary cortisol (sAA) values of Figure 2a within the time course t1‐t4. Note. P‐TSST: Placebo‐TSST. T1 = baseline, t2 = +25 min, t3 = +40 min, t4 = +60 min. Table S2. Alpha‐Amylase values of Figure 2b within the time course t1‐t4. Note. P‐TSST: Placebo‐TSST. T1 = baseline, t2 = +25 min, t3 = +40 min, t4 = +60 min. Table S3. Mean values of control stimuli of the Cue‐Reactivity Paradigm. Note. Control stimuli: online buying‐shopping stimuli. Table S4. Correlational Analysis of stress condition, implicit cognition measures and Craving response by the Cue‐Reactivity Paradigm. n = 70. Note. TSST condition: TSST vs. p‐TSST, Subjective urge: CRP urge; Subjective arousal: CRP arousal, Subjective valence: CRP valence, **P < 0.01. [file ADD-120-2067-s001.docx]

Supplemental Material

**A1 Exclusion criteria**

Exclusion criteria were physical or mental disorders that would affect the completion of the questionnaires and paradigms. In addition, it was ensured that there were no current suicidal thoughts, and no substance use other than tobacco. Subjects were required to be between the age of 18 and 65, should possess sufficient German language skills and had to be a clinically relevant case for p-SNU or be able to be classified as individual with non-problematic use indicated as control participant to participate in the study.

**A2 Implicit Association Test**

In the Implicit Association Test, participants are asked to assign attribute concepts to SN-related pictures or control pictures based on given instructions. For the picture-related attribute concepts, ‘positive’ and ‘negative’ pictures from the International Affective Picture System (IAPS) are used. An example of a positive picture would be a happy couple, while a crying child represents a negative-associated picture. A bipolar structure was chosen as previous research (1) has shown that the choice of ‘positive’ vs. ‘negative’ as attribute concepts performs better in alcohol-related IATs than other variants (e.g. ‘positive’ vs. ‘neutral’ or ‘negative’ vs. ‘neutral’). Participants are asked to categorize pictures into target concepts (‘social networks’ vs. ‘online shopping’) and attribute concepts (‘positive’ vs. ‘negative’) as quickly as possible by pressing one of two keys on the response pad. Within each concept category, ten images are presented in a randomized order. It is assumed that people with p-SNU respond faster to congruent pairings (‘SN-related stimuli or positive’ vs. ‘control stimuli or negative’) than to incongruent pairings (‘control stimuli or positive’ vs. ‘SN-related stimuli or negative’). The instructions alternate in different rounds as follows:

Round 1 and 2: The target (‘SN-related stimuli’ vs. ‘control stimuli) and attribute (‘positive imagery’ vs. ‘negative imagery’) concepts are introduced and practiced.

Rounds 3 and 4: The target and attribute concepts are combined in an SN-congruent way (‘SN-related or positive’ for one response key and ‘control stimuli or negative’ for the other key).

Round 5: The response keys for the target concept are swapped and practiced again.

Rounds 6 and 7: Target and attribute concepts are combined in an SN-incongruent manner (‘control stimuli or positive’ vs. ‘SN-related or negative’).

**A3 Dot Probe Paradigm**

In the Dot Probe Paradigm, participants are presented with a black background on the desktop, on which a central fixation cross is then shown for 500ms at the start of each trial. This is followed by the presentation of an SN-related stimuli and a control stimulus (on the left and right side of the screen respectively) for 200ms. After the image has disappeared, a white dot (Arial, size 50, on a black background) is presented to replace the position of either the SN-related stimuli or the control stimuli. The subjects must then indicate the position of the dot (left or right) by pressing one of two response keys on the response pad as quickly and accurately as possible. The dot is displayed until the participant has pressed one of the response buttons. Each pair of pictures is presented four times, with the side (left/right) and the position of the dot probe (left/right) being counterbalanced, resulting in 160 presentation attempts. Only the pairs with SN-related images are analyzed. Response latency (in ms) is recorded as the dependent variable and trials with response errors and response times <100 ms or >1000 ms are excluded.

**References**

1. Houben K, Havermann RC, Wiers RW. Learning to dislike alcohol: Conditioning negative implicit attitudes toward alcohol and its effect on drinking behavior. Psychopharmacology. 2010;211:79-86.

**Tables**

Table S1. Salivary cortisol (sAA) values of Fig. 2a within the time course t1-t4.

|  | p-TSST |  |  | TSST |  |
| --- | --- | --- | --- | --- | --- |
|  | *M(SD)* | *95% CI* |  | *M(SD)* | *95% CI* |
|  | Control Group | | | | |
| t1 | 3.21(1.99) | 2.57, 3.98 | t1 | 3.26(1.93) | 2.61-3.95 |
| t2 | 3.01(1.72) | 2.49, 3.70 | t2 | 4.26(2.41) | 3.47-5.13 |
| t3 | 2.90(1.53) | 2.42, 3.50 | t3 | 6.31(5.34) | 4.66-8.28 |
| t4 | 2.67(1.26) | 2.26, 3.14 | t4 | 6.02(5.15) | 4.45-7.95 |
|  |  |  |  |  |  |
|  | p-SNU Group | | | | |
| t1 | 2.73(1.23) | 2.35, 3.14 | t1 | 2.92(2.17) | 2.21-3.81 |
| t2 | 2.59(1.32) | 2.19, 3.04 | t2 | 3.71(2.62) | 2.87-4.73 |
| t3 | 2.62(1.28) | 2.20, 3.08 | t3 | 5.53(3.84) | 4.34-7.01 |
| t4 | 2.40(1.17) | 0.73, 1.62 | t4 | 4.72(3.05) | 2.04-3.89 |
|  |  |  |  |  |  |

Note. P-TSST: Placebo-TSST. T1=baseline, t2=+25 min, t3=+40min, t4=+60min.

Table S2. Alpha-Amylase values of Fig. 2b within the time course t1-t4.

|  | p-TSST |  |  | TSST |  |
| --- | --- | --- | --- | --- | --- |
|  | *M(SD)* | *95% CI* |  | *M(SD)* | *95% CI* |
|  | Control Group | | | | |
| t1 | 121.64(74.89) | 98.18, 148.52 | t1 | 126.01(107.83) | 92.12, 166.25 |
| t2 | 131.03(87.26) | 102.27, 164.48 | t2 | 217.23(184.28) | 157.14, 286.91 |
| t3 | 132.87(101.25) | 99.43, 171.54 | t3 | 121.41(90.97) | 89.99, 154.86 |
| t4 | 127.85(95.86) | 97.43, 166.09 | t4 | 138.78(108.97) | 103.54, 179.83 |
|  |  |  |  |  |  |
|  | p-SNU Group | | | | |
| t1 | 178.29(174.92) | 128.14, 237.29 | t1 | 148.74(146.05) | 99.34, 202.05 |
| t2 | 214.42(297.80) | 140.24, 315.47 | t2 | 206.16(148.17) | 155.75, 258.04 |
| t3 | 142.65(181.38) | 99.76, 204.71 | t3 | 137.67(115.36) | 99.32, 176.34 |
| t4 | 144.31(171.06) | 100.05, 200.03 | t4 | 130.59(115.36) | 91.93, 172.21 |
|  |  |  |  |  |  |

Note. P-TSST: Placebo-TSST. T1=baseline, t2=+25 min, t3=+40min, t4=+60min.

Table S3. Mean values of control stimuli of the Cue-Reactivity Paradigm

|  | **p-SNU**  (*n* = 71) | | **Control group**  (*n* = 64) | | **Group Comparison** |
| --- | --- | --- | --- | --- | --- |
|  | *M (SD)* | *95% CI* | *M (SD)* | *95% CI* |  |
| *Control stimuli* | | | | | |
| Subjective arousal | 2.30 (.97) | 2.09, 2.53 | 1.74 (.82) | 1.55, 1.95 | *t*(131)=-3.57, *p*<.001 │*d*│=.62 |
| Subjective urge | 2.16 (.99) | 1.94, 2.39 | 1.58 (.79) | 1.39, 1.80 | *t*(131)=-3.70, *p*<.001, │*d*│=.64 |
| Subjective valence | 2.92 (.67) | 2.75, 3.10 | 2.79 (.76) | 2.62, 2.97 | *t*(131)=-.96, *p*=.337, │*d*│=-.17 |
|  |  |  |  |  |  |

Note. Control stimuli: online buying-shopping stimuli.

Table S4. Correlational Analysis of stress condition, implicit cognition measures and Craving response by the Cue-Reactivity Paradigm. *n*=70

|  | TSST  condition | Attentional Bias | Implicit Associations | Subjective arousal | Subjective  urge |
| --- | --- | --- | --- | --- | --- |
|  |  |  |  |  |  |
| Attentional Bias | .076 |  |  |  |  |
| Implicit Associations | -.057 | .194 |  |  |  |
| Subjective arousal | .143 | -.224 | -.086 |  |  |
| Subjective urge | .089 | -.235* | -.082 | .886** |  |
| Subjective valence | .081 | -.190 | -.070 | .603** | .557** |

Note. TSST condition: TSST vs. p-TSST, Subjective urge: CRP urge; Subjective arousal: CRP arousal, Subjective valence: CRP valence, ***P*<.01.
